# Fecal Transplantation Treatment of Antibiotic-Induced, Noninfectious Colitis and Long-Term Microbiota Follow-Up

**DOI:** 10.1155/2014/913867

**Published:** 2014-11-19

**Authors:** Reetta Satokari, Susana Fuentes, Eero Mattila, Jonna Jalanka, Willem M. de Vos, Perttu Arkkila

**Affiliations:** ^1^Department of Veterinary Biosciences, University of Helsinki, P.O. Box 66, 00014 Helsinki, Finland; ^2^Laboratory of Microbiology, Wageningen University, Dreijenplein 10, 6703 HB Wageningen, The Netherlands; ^3^Department of Infectious Diseases, Helsinki University Central Hospital, P.O. Box 348, 00029 Helsinki, Finland; ^4^Haartman Institute, University of Helsinki, P.O. Box 21, 00014 Helsinki, Finland; ^5^Department of Gastroenterology, Helsinki University Central Hospital, P.O. Box 372, 00029 Helsinki, Finland

## Abstract

Fecal microbiota transplantation (FMT) is an effective treatment for recurrent *Clostridium difficile* infection (CDI) and is considered as a treatment for other gastrointestinal (GI) diseases. We followed up the relief of symptoms and long-term, over-a-year microbiota stabilization in a 46-year-old man, who underwent FMT for antibiotic-induced, non-CDI colitis nine months after being treated for CDI by FMT. Fecal and mucosal microbiota was analyzed before the second FMT and during 14 months after FMT by using a high-throughput phylogenetic microarray. FMT resolved the symptoms and restored normal GI-function. Microbiota analysis revealed increased bacterial diversity in the rectal mucosa and a stable fecal microbiota up to three months after FMT. A number of mucosa-associated bacteria increased after FMT and some of these bacteria remained increased in feces up to 14 months. Notably, the increased bacteria included *Bifidobacterium* spp. and various representatives of *Clostridium* clusters IV and XIVa, such as *Clostridium leptum*, *Oscillospira guillermondii*, * Sporobacter termitidis*, * Anaerotruncus colihominis*, * Ruminococcus callidus*, * R. bromii*, * Lachnospira pectinoschiza*, and *C. colinum*, which are presumed to be anti-inflammatory. The presented case suggests a possible role of microbiota in restoring and maintaining normal GI-functionality and improves our knowledge on the etiology of antibiotic-induced, noninfectious colitis.

## 1. Introduction

The intestinal tract harbors an extremely diverse microbiota, which is crucial in maintaining immunological and physiological homeostasis of the mucosa [1]. Dysbiosis of microbiota (aberrant composition) can lead to loss of normal, regulatory immune effects in the gut mucosa, and dysbiotic microbiota has been associated with a number of inflammatory and immune-mediated diseases [2, 3]. A recent hypothesis presents that, after epithelial insult by a pathogen or an injury, specific symbiotic bacteria are needed to restore mucosal homeostasis back to a basal level by ceasing the acute inflammation [3]. In support of the hypothesis, a community of 17 intestinal strains attenuated intestinal inflammation by stimulating regulatory T cells in a mouse colitis model [4]. Fecal microbiota transplantation (FMT) is effective in treating recurrent* Clostridium difficile* infection (CDI) [5, 6], and it is increasingly considered also for the treatment of noninfectious colitis [7]. This report describes treatment of antibiotic-induced, noninfectious colitis by FMT nine months after the first FMT treatment for CDI and the stabilization of intestinal microbiota in a 46-year-old man during 14 months after the second FMT.

## 2. Materials and Methods

### 2.1. The Case Presentation

The patient was a 46-year-old man with history of hypertension and ventricular extrasystolia, which were being controlled by medication. He had no known allergies, and he had travelled only in Western Europe. He smoked. He had had several sinusitis episodes. Two years before the onset of refractory* Clostridium difficile* infection, he had septic infection due to the axillar bursitis. His uncle has Crohn's disease.

The patient had* Clostridium difficile* infection (CDI), after amoxicillin clavulanate treatment for otitis media. CDI was verified by cultivation of* C. difficile* and positive toxin test of feces (VIDAS* C. difficile* Toxin A & B CDAB, BioMerieux, France). No resolution of symptoms was achieved with oral metronidazole (400 mg thrice a day) or vancomycin (first 120 mg four times a day and later 250 mg four times a day). Because of refractory situation, patient was hospitalized and the treatment was switched to meropenem (1 g thrice a day intravenously) and rifaximin (400 mg twice a day). In addition, he got one dose of immunoglobulin 27.5 g intravenously. After six days of treatment, the patient was discharged with ten days of rifaximin (400 mg twice a day) treatment. Three days after stopping the rifaximin treatment, the symptoms reappeared including 10 diarrhea episodes per day. The patient restarted rifaximin treatment and continued it until colonoscopy and FMT were done three weeks later. Mild nonspecific proctitis was found in colonoscopy. The histology showed postinfectious inflammation and no typical signs of chronic inflammatory bowel disease. FMT was performed by infusing fecal suspension into the caecum. The diarrhea disappeared within two days after FMT.

Seven months after FMT, the patient got clarithromycin for sinusitis and diarrhea reappeared. Symptoms were not as severe as earlier; that is, he had 5-6 diarrhea episodes per day and urgency of defecation. No* C. difficile* or toxins were found in feces by cultivation or toxin tests, respectively. The symptoms did not resolve spontaneously during 8 weeks after the cessation of clarithromycin. Colonoscopy was reassessed to exclude chronic inflammatory bowel disease. The macroscopic and microscopic findings were similar as seen previously, that is, mild inflammation. Fecal calprotectin was also tested and was 12 *μ*g/g. The patient received the second FMT and thereafter he has had no GI-symptoms (two and a half years by the time of submitting this report). The patient had three antibiotic courses during the follow-up period between 10 and 12 months after the second FMT.

### 2.2. The Donor and Fecal Microbiota Transplantation (FMT)

The donor of fecal material in both transplantations was the patient's 35-year-old wife, who was pregnant at the time of the second FMT. She had not received antimicrobial therapy for the past 6 months and did not have any intestinal symptoms. She was tested by the protocol for fecal donors as described previously [5]. Thirty grams of the donor's feces were suspended into 150 mL of tap water and the suspension was infused into the caecum through colonoscopy as described previously [5].

### 2.3. Samples and Microbiota Analysis

**Figure 1:**
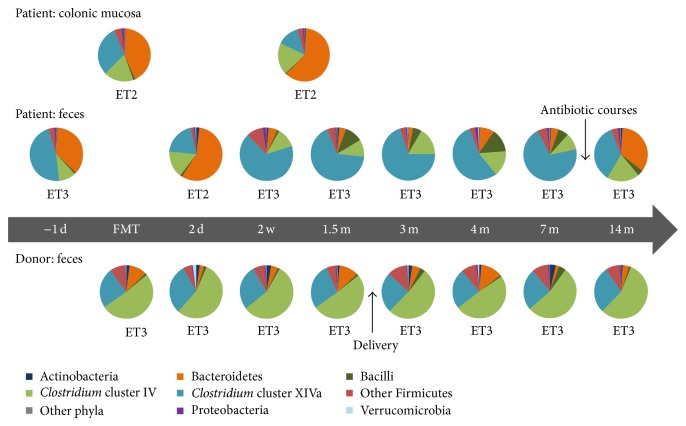
Microbiota composition at the phylum level and bacterial genus level enterotype (ET) status before FMT and during the 14-month follow-up period.

Rectal biopsies were taken from the patient at the time of the second FMT and one month later in proctoscopy and stored in −80°C until further processing. The patient and donor collected fecal samples after the second FMT according to the sampling scheme presented in Figure 1. The fecal samples were placed in a home freezer at −20°C immediately after defecation and stored in the home freezer for maximally 4 months until transfer to laboratory for further analysis. Six healthy adult volunteers (3 males and 3 females, mean age 67 years), who underwent diagnostic colonoscopy and were found to have a healthy intestine, were included as a comparison group for mucosa-associated microbiota. DNA extraction was done as described previously by using mechanical disruption of bacterial cells by bead-beating followed by DNA purification [8]. Microbiota analysis was done by using the HITChip high-throughput bacterial phylogenetic microarray as described previously [8–11].

### 2.4. Ethical Considerations

The study was approved by the Ethics Committee of Hospital District of Helsinki and Uusimaa (HUS), Finland (Dnro HUS 124/13/03/01/11). The patient was informed about the experimental nature and possible risks of FMT. Written informed consent was obtained from the patient and donor for publication of this case report. The healthy volunteers who donated biopsies signed informed consent.

## 3. Results

### 3.1. Resolution of GI-Symptoms by FMT

The patient's recurrent* Clostridium difficile* infection was successfully treated by FMT. Seven months later, he got antibiotic-induced diarrhea, which was not as severe as earlier. Also, no* C. difficile* or toxins were found in feces. The symptoms did not resolve spontaneously within eight weeks after stopping the antibiotic. Colonoscopy excluded inflammatory bowel disease, but mild inflammation was observed. The patient received FMT again and the GI-symptoms were resolved within few days. The patient had three antibiotic courses during the follow-up period between 10 and 12 months after the second FMT, but these antibiotic treatments did not induce diarrhea.

### 3.2. Fecal Microbiota of the Donor

**Figure 2:**
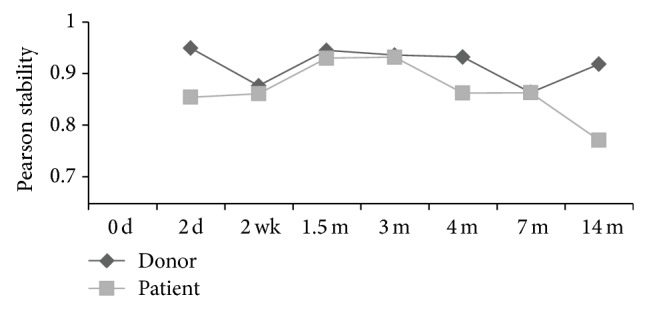
Stability of the fecal microbiota determined as Pearson correlation of the microbiota profiles between the subsequent sampling points.

**Figure 3:**
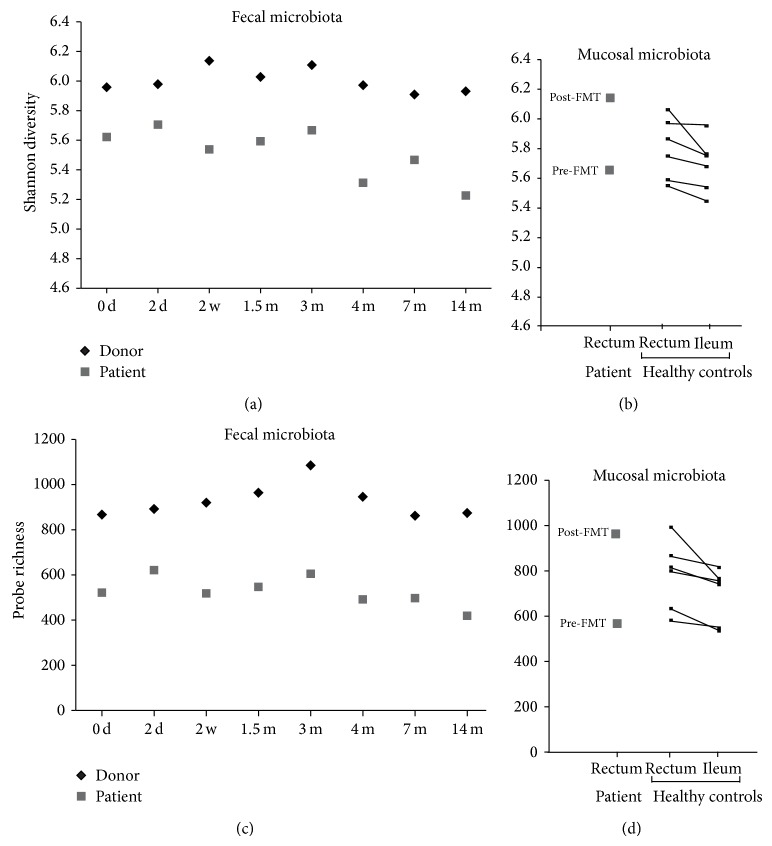
Diversity and richness of microbiota in the fecal samples (a and c) and in mucosal biopsies from the patient and six healthy controls (b and d). 0 d: pre-FMT sample; 2 wk to 14 mo: post-FMT samples. Line connects the rectal and ileal samples from the same individual.

In general, fecal microbiota of the donor was found to be stable during the 14-month follow-up period, but a decrease in stability was observed between 2-day and 2-week and 4- and 7-month samples, that is, approximately one month before and three months after the delivery, respectively (Figure 2). The most predominating phylum was Firmicutes, where* Clostridium* clusters IV and XIVa and uncultured Clostridiales were the largest groups (Figure 1). Phylum Bacteroidetes constituted 2–13% and Actinobacteria and Proteobacteria less than 4% of the total microbiota in the donor (Figure 1). In the enterotype (ET) profiling, the donor's fecal microbiota represented ET3, that is, the* Ruminococcus* enterotype (Figure 1). The diversity and richness of fecal microbiota were higher in the donor than in the patient throughout the follow-up period (Figure 3).

### 3.3. Mucosal and Fecal Microbiota of the Patient during 14 Months after the FMT

Intestinal microbiota composition was followed up with frequent sampling during 14 months after the FMT. In the patient, a shift from a Bacteroidetes-rich fecal microbiota to a microbiota, where Firmicutes constitute over 87% of the bacterial community, was observed from two weeks after FMT onwards (Figure 1). Particularly, the relative proportion of* Clostridium* cluster XIVa increased by 20% in feces after FMT. However, in mucosa, the relative abundance of the phylum Bacteroidetes increased and Firmicutes decreased after FMT. Fecal microbiota two days after FMT resembled the pre-FMT microbiota and may reflect an adaptation stage. Also, fecal microbiota enterotype (ET) profiling analysis showed a shift at 2 days after FMT. Fecal microbiota represented enterotype 3 (ET3), that is, the* Ruminococcus* enterotype at all other sampling points except at 2 days after FMT. In contrast, mucosal microbiota represented enterotype 2 (ET2), that is, the* Prevotella* enterotype (Figure 1). Consistently, both the phylum level and enterotype profiling, that is, genus level analysis, showed that the composition of mucosa-associated microbiota is different from that of the fecal microbiota. The patient's fecal microbiota was stable from 2 weeks to 3 months after FMT, but then stability decreased slightly between 3 and 7 months and more notably between 7 and 14 months (Figure 2). From 7- to 14-month fecal samples, a shift back to a Bacteroidetes-rich fecal microbiota was seen (Figure 1). The shift cooccurred with the administration of three antibiotic courses between these sampling points.

Further, the analysis of mucosa-associated microbiota revealed a marked increase in the diversity and richness of bacterial species in post-FMT rectal biopsy as compared to the pre-FMT biopsy (Figure 3). The increase in diversity and richness in the patient's rectal mucosa exceeded the interindividual variability of rectal biopsies from six healthy controls (Figure 3). The increase in diversity also exceeded intraindividual variability between ileal and rectal biopsies from the same individual (Figure 3). The results indicate that the increase in diversity and richness of the patient's mucosal microbiota is beyond the natural variability. Thus, the mucosal microbiota of the patient seemed to be strongly affected by FMT. However, in the luminal contents (feces), the increase in bacterial diversity and richness was modest (Figure 3). Interestingly, the post-FMT biopsy of the patient clustered with fecal samples of the donor in hierarchical clustering of the microbiota profiles (Supplementary Figure  1 available online at http://dx.doi.org/10.1155/2014/913867). A number of bacterial groups in the mucosa showed either a decrease or an increase in their relative abundance after FMT (Table 1). Most of these bacterial groups (46 out of 49) showed a similar difference between the donor and the pre-FMT patient fecal samples. Further, specific bacterial groups in post-FMT mucosal sample showed the same direction of change in their relative abundance in the fecal sample at 14 months after FMT and during the follow-up period from 2 weeks to 7 months (in average) as compared to the pre-FMT feces (Table 1). The increased bacterial groups include* Bifidobacterium* spp. (Actinobacteria),* Clostridium leptum et rel.*,* Oscillospira guillermondii et rel.*,* Sporobacter termitidis et rel.*,* Anaerotruncus colihominis et rel.*,* Ruminococcus callidus et rel.*,* R. bromii et rel.* (*Clostridium* cluster IV),* Dialister* spp. (*Clostridium* cluster IX),* Lachnospira pectinoschiza* et rel.,* C. colinum et rel.* (*Clostridium* cluster XIVa), uncultured Clostridiales I, and* Oxalobacter formigenes et rel*. (Proteobacteria) (Table 1). We assume that these bacterial groups have established themselves stably from the donor's microbiota or have successfully and stably recovered from the patient's own microbiota after FMT. On the other hand, four groups belonging to Bacteroidetes and* Clostridium ramosum et rel*. (*Clostridium* cluster XVIII) decreased after FMT both in mucosa and in feces.

## 4. Discussion

The case described herein presents an expansion of the use of FMT to treat antibiotic-induced, non-CDI colitis. FMT restored normal GI-function and the patient has not had GI-symptoms thereafter, that is, two and a half years at the time of writing this report.

Interestingly, the patient did not seem to have perturbed fecal microbiota prior to FMT. The relative proportion of Bacteroidetes was rather high, but within the normal variation in healthy Europeans adults [9]. Also, the microbiota composition of the donor was typical to healthy adults [9] and showed high stability during the last month of pregnancy and three months postpartum, which is in line with the results from a recent study [12]. In the patient, the proportion of Bacteroidetes still increased two days after the FMT, but later a clear shift to a Firmicutes-dominating microbiota was observed. It seems that a period ranging from few days to two weeks is needed before the establishment of post-FMT microbiota. The temporary shift of the microbiota enterotype at 2d after FMT also indicates a transition period. At the phylum level, fecal microbiota was relatively stable from two weeks up to three months after FMT. Recently, a stable phylum level microbiota composition was reported in three patients, who were followed up for 3-4 months after FMT for recurrent* C. difficile* infection [13]. In our patient, the diversity of microbiota decreased and the total microbiota was less stable after three months after FMT. The result indicates that microbiota evolution may continue for a considerable period of time after FMT and underlines the need for long-term post-FMT follow-up studies of microbiota stabilization.

In the patient, fecal and mucosal microbiota showed different compositions at both bacterial phylum and genus levels. In the enterotype profiling, fecal microbiota was found to be enterotype 3 (ET3) and mucosal microbiota enterotype 2 (ET2). The three enterotypes are each characterized by a discriminating genus,* Bacteroides* (ET1),* Prevotella* (ET2), or* Ruminococcus* (ET3), whose abundance correlates with the abundance of other genera [11]. Thus, the enterotypes are driven by groups of bacteria that together contribute to the preferred microbiota composition, which is a result of adaption to the particular environment. Interestingly, the bacterial diversity and richness were higher in the mucosal biopsies as compared to feces. Similarly, higher bacterial richness was observed in the mucosa as compared to matched fecal samples in a recent study comprising four individuals [14]. Clearly, intestinal lumen and mucosa represent different environments and, consequently, harbor different microbiota.

We observed a shift back to a Bacteroidetes-rich fecal microbiota in the patient at 14 months after FMT. The shift was associated with the administration of three antibiotic courses, which, however, did not induce diarrhea this time. Thus, at the phylum level composition, the patient's microbiota at 14 months resembled microbiota before FMT, but apparently he maintained some key bacterial species that could support gut functionality even when challenged by antibiotics. Indeed, we observed a drastic increase in the diversity and richness of mucosal microbiota after FMT indicating either the strong engraftment of donor's microbiota or the recovery of patient's own microbiota particularly in the mucosal lining. Specific mucosa-associated bacteria, which increased in abundance, showed an increase also in feces up to 14 months after FMT. The increased bacterial groups include* Bifidobacterium* and* Dialister* spp., bacteria related to* Clostridium leptum*,* Oscillospira guillermondii*,* Sporobacter termitidis*,* Anaerotruncus colihominis*,* Ruminococcus callidus*,* R. bromii*,* Lachnospira pectinoschiza*,* C. colinum*, and* Oxalobacter formigenes*, as well as uncultured Clostridiales I. We assume that these bacterial groups have established themselves stably after FMT and may have a crucial role in maintaining gut homeostasis. Previously,* Bifidobacterium*,* C. leptum*,* Sporobacter*,* Oscillospira*,* Anaerotruncus*,* R. bromii*,* Dialister*, and uncultured Clostridiales have been found to be increased in healthy individuals as compared to inflammatory bowel disease (IBD) patients [10, 15]. Further, a combination of 17 strains of intestinal bacteria, belonging to the genera* Ruminococcus*,* Anaerotruncus*,* Lachnospiraceae*,* Clostridium*,* Eubacterium*, and* Blautia*, has been shown to attenuate inflammation in a mouse colitis model by stimulating regulatory T cells [4]. As an exception,* O. formigenes* has no reported anti-inflammatory effects but is associated with a reduced risk of calcium oxalate stone disease [16]. Collectively, our patient showed constantly increased levels of intestinal bacteria, which seem to have anti-inflammatory effects and possibly ceased the mild, on-going inflammation.

In the presented case, FMT resolved the symptoms in an antibiotic-induced, noninfectious colitis. The post-FMT bacterial community restored normal GI-function possibly by ceasing the mild, on-going inflammation. The microbiota analysis suggests that specific members of microbiota may have a role in restoring and maintaining normal GI-functionality. Interestingly, the 17 strains in the study by Atarashi et al. [4] exerted an anti-inflammatory effect as a community, but not as individual strains. Thus, it seems that an assembly of bacterial species, rather than a specific single species, is essential in preventing or treating intestinal diseases. Today, FMT is the most straightforward way of introducing the necessary combination of bacteria to a patient, but there is an increasing need to identify the crucial bacterial species that are able to control infection or inflammation in the intestine. The first two cases on the use of an “artificial stool” consisting of pure cultures of intestinal bacterial have shown promising results in the treatment of recurrent CDI [17]. Clinical FMT-studies linked with microbiota analysis can greatly advance these efforts and improve our understanding of the etiology of diseases, where dysbiosis of the microbiota is considered to play a role.

## 5. Conclusions

The antibiotic-induced, noninfectious colitis of a 46-year-old man was successfully treated with FMT. The results suggest a possible role of intestinal microbiota and its specific members in restoring and maintaining normal GI-functionality.

## Supplementary Material

Supplementary figure 1. Hierarchical clustering based of the HITChip phylogenetic microarray profiles of the patient (P) and donor (D) fecal samples and patient biopsy samples (P_B). The samples are coded by the time of collection, where 0 represents the pre-FMT sample and d, wk and m represent days, weeks or months after the FMT, respectively. The color intensity represents microarray probe signal level, which corresponds to the bacterial abundance in the sample. The highest phylogenetic level of probes' specificity is depicted on the side of the profiles. Pearson correlation and Ward's clustering method were used.

## Figures and Tables

**Table 1:** Mucosal bacteria with >2-fold change in relative abundance after FMT and constituting >0.01% of the microbiota.

Phylum/cluster	Increased bacterial groups in colonic mucosa^1^	Fold-change B0 to B1	Increased in feces F14?m and F2?w–7?m^3^
Actinobacteria	*Bifidobacterium *spp.	2.2	Yes

Bacteroidetes	*Prevotella melaninogenica et rel. *	5.6	No
	*Prevotella oralis et rel. *	5.7	No

Firmicutes/*Clostridium* cl. IV	*Clostridium leptum et rel. *	2.5	Yes
	*Oscillospira guillermondii et rel. *	4.8	Yes
	*Sporobacter termitidis et rel. *	2.7	Yes
	*Anaerotruncus colihominis et rel. *	3.4	Yes
	*Ruminococcus callidus et rel. *	4.1	Yes
	*Ruminococcus lactaris et rel. *	15.4	No
	*Ruminococcus bromii et rel. *	12.0	Yes
	*Subdoligranulum variabile et rel. *	7.9	No

Firmicutes/*Clostridium* cl. IX	*Dialister *spp.	3.6	Yes

Firmicutes/*Clostridium* cl. XIVa	*Lachnospira pectinoschiza et rel. *	2.3	Yes
	*Ruminococcus lactaris et rel. *	15.4	No
	*Butyrivibrio crossotus et rel. *	2.5	No
	*Clostridium colinum et rel. *	3.3	Yes

Uncult. Clostridiales	Uncult. ClostridialesI	10.2	Yes

Proteobacteria	*Oxalobacter formigenes *	3.5	Yes

Phylum/cluster	Decreased bacterial groups in colonic mucosa^2^	Fold-change B0 to B1	Decreased in feces F14 m and F2 w–7 m^3^

Bacteroidetes	*Alistipes et rel. *	2.0	No
	*Bacteroides stercoris et rel. *	2.4	No
	*Bacteroides vulgatus et rel. *	9.6	Yes
	*Bacteroides fragilis et rel. *	2.1	Yes
	*Bacteroides ovatus et rel. *	3.4	Yes
	*Bacteroides intestinalis et rel. *	3.4	No
	*Bacteroides plebeius et rel. *	2.5	No
	*Prevotella tannerae et rel. *	6.2	Yes
	*Tannerella et rel. *	2.4	No
	*Parabacteroides distasonis et rel. *	4.0	No
	Uncult. Bacteroidetes	5.9	No

Firmicutes/*Clostridium* cl. I	Clostridia	5.8	No

Firmicutes/*Clostridium* cl. XIVa	*Coprococcus eutactus et rel. *	2.4	No
	*Dorea formicigenerans et rel. *	4.0	No
	*Eubacterium rectale et rel. *	2.0	No
	*Ruminococcus gnavus et rel. *	7.0	No
	*Ruminococcus obeum et rel. *	4.4	No
	*Clostridium nexile et rel. *	5.3	No
	Outgr. *Clostridium* cl. XIVa	6.2	No

Firmicutes/*Clostridium* cl. IX	*Veillonella *spp.	2.7	No
	*Megasphaera elsdenii et rel. *	3.4	No

Firmicutes/*Clostridium* cl. XI	*Clostridium difficile et rel. *	23.1	No

Firmicutes/*Clostridium* cl. XVI	*Bulleidia moorei et rel. *	2.1	No
	*Eubacterium cylindroides et rel. *	2.2	No

Firmicutes/*Clostridium* cl. XVIII	*Clostridium ramosum et rel. *	8.1	Yes

Firmicutes/Bacilli	*Streptococcus bovis et rel. *	2.3	No
	*Streptococcus intermedius et rel. *	2.3	No
	*Streptococcus mitis et rel. *	2.1	No

Proteobacteria	*Sutterella wadsworthia et rel. *	2.7	No
	*Escherichia coli et rel. *	3.7	No
	*Klebsiella pneumonia et rel. *	2.2	No

^1^Higher abundance also in the donor's feces as compared to the patient's pre-FMT feces; ^2^lower abundance also in the donor's feces as compared to the patient's pre-FMT feces, with the exception of* C. eutactus et rel., E. rectale et rel.,* and *S. bovis et rel*.; ^3^increased/decreased abundance in 14 mo and in 2 w to 7 mo (average) post-FMT samples as compared to the pre-FMT fecal sample; Uncult.: uncultured; cl.: cluster; Outgr.: outgrouping.
