# Supplementary figures and images for: Fecal Transplantation Treatment of Antibiotic-Induced, Noninfectious Colitis and Long-Term Microbiota Follow-Up
Source: Case Rep Med. 2014 Nov 19;2014:913867. doi: 10.1155/2014/913867 (PMC4274837; doi:10.1155/2014/913867)

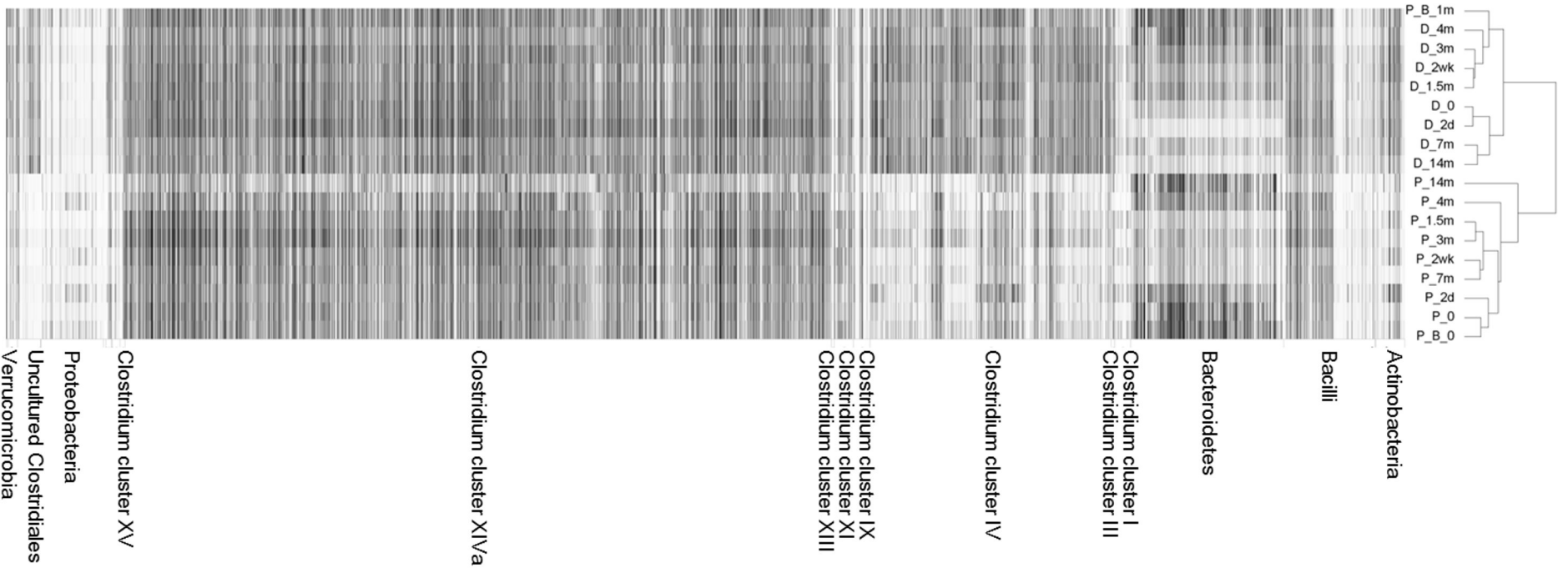

Supplement: Supplementary file 1 — Supplementary figure 1. Hierarchical clustering based of the HITChip phylogenetic microarray profiles of the patient (P) and donor (D) fecal samples and patient biopsy samples (P_B). The samples are coded by the time of collection, where 0 represents the pre-FMT sample and d, wk and m represent days, weeks or months after the FMT, respectively. The color intensity represents microarray probe signal level, which corresponds to the bacterial abundance in the sample. The highest phylogenetic level of probes' specificity is depicted on the side of the profiles. Pearson correlation and Ward's clustering method were used. [file 913867.f1.pdf]
